# Indents

**Published:** 1910-05-15

**Authors:** 


### INDENTS.

**☞ Brief Articles of Technical or Scientific Value will receive notice and comment. Contributions invited.**

*Paraform as an Obtundent.* Last March I gave a short communication relating to my experience in the use of paraform. Since then I have used it in forty additional cases in 28 patients, and I append a tabulated statement, containing particulars of each tooth treated, so that a correct judgment may be formed as to the use of the drug.

I would like to point out that I have included *all* cases in the statement, and I have made careful *notes at the time* of each stage, so that I think I may claim impartiality in my report.

I have avoided the use of the drug in all really deep cavities and have selected only those which were *especially sensitive*, and in some cases they were the type of shallow hyper-sensitive cavity which, as a rule, is the most difficult to treat. I have not had any case, so far, of death of a pulp, nor any case where the pulp has had to be treated subsequently, and I think the fact (observed in more than one case) that quite localised anaesthesia of part of the dentine may be obtained proves that the effect is there and not in the pulp if proper precautions are taken.

Another point to which I would like to call your attention is that many of these patients were of a rather neurotic type, and therefore I think the results are all the more gratifying. I have not used it in any very young children.

I have recently been trying another mixture with G. P., containing 20 per cent. paraform (kindly supplied by Mr. Campion), but I think, on the whole, 5 per cent. is the most useful proportion.

An analysis of the results shows that of the forty teeth treated, in 18 there was complete anaesthesia, in 14 there was almost complete anaesthesia, in five there was only a slight effect, and in 3 cases no result, two of the last three cases were in the same mouth.—J. A. Woods, in Jany. 1910 *Dental Record*.

*System in Cavity Preparation.* I have long followed the division into definite steps, of the operation of preparing cavities, differing from the essayist only in the order of their execution.

First. Open the cavity, establishing the outline.

Second. Remove all *softened* dentine.

Third. Shape the cavity for resistance, retention and convenience.

Fourth. Finish enamel walls and bevel cavo-surface angles.

Fifth. Dress and clean the cavity.

No matter what are the operator's views regarding extension for prevention or the *shape* best fitted to fulfill the requirements as noted, he *must do these things.* If he proceeds in the order suggested by either of us, his labors will be lightened and his patient subjected to a minimum of discomfort. I must agree that extension for prevention, when clearly understood, as Dr. Black sees it, does not mean a ruthless destruction of good tooth structure, but when I view some of the results I have seen in many instances, where an effort has been made in this direction, I do not wonder that the principle is condemned, for about the only thing accomplished *was* the destruction of good tooth substance.

In the preparation of cavities, especially classes second and third, there are two things we must always see clearly: one is the mechanics, and the other, the prophylactic result to be obtained in the completed filling. In the first we must study the structure and architecture of the tooth in the field under operation. How much is it weakened by the progress of the caries? This can only be decided after the weak enamel walls have been cut away and all softened dentine removed. How must our cavity be formed to fulfill its requirements and leave the tooth that remains with the greatest strength possible under the conditions? In deciding this we must have in mind the second consideration—what is to be the form and contour of the completed filling in order that it may do its share in establishing the proper contact and correct interproximal space which opens into wide embrasures.—Dr. T. E. Weeks in *Brief.*

*Matrix.* If I desire to fill the disto-occlusal surface of a left lower first molar, for instance, I take an extracted lower molar of that side, cut off the roots a little way below the gingival border, make a plaster cone and wax the amputated crown on the apex of the cone with the distal surface of the crown forming the apex of the whole. I then make a matrix of the same in sand and run a zinc die. To make my matrix I have only to place my matrix metal on a piece of lead, place my die on the same, and give it a few raps with a hammer, and I have a perfectly contured matrix of the distal surface of the left lower molars. One die will answer for that surface of all the left lower molars and with your pliers you can in a few moments, make any changes that be necessary. A set of about six (6) dies will be all that are necessary for all the teeth, and they will last a lifetime. The edges of the matrix must be shaped up and smoothed by using a carborundum stone. This matrix will give you the perfect contour of the class of tooth you wish to use it for.—Dr. Milliken in *Brief.*

*The Matrix in Gold Fillings.* Dr. Black does not agree with the use of the matrix, but I have learned a method of using gold in which I successfully use the matrix. I have demonstrated it to a good many dentists, and so far as I know, all who have adopted that method have never given it up. What is the critical feature of all gold filling? Certainly the margin. A filling that is defective in some particulars, if it has a perfect margin, will be fairly successful. I have said before that the features which lead to failure in gold fillings may often be microscopic. I do not think it is within the range of human skill to pack rolled gold or ribbon gold so tight over the margin of the filling that one can be absolutely certain that there is no microscopic space left there. I have described this method in some of the American journals. I don't know to what extent it has been adopted here, but so far as I have heard from those who have adopted it, they have always expressed the greatest satisfaction with it. I am sometimes able to fill the cavity one-third or a half full before resorting to this particular method, but go as far as you can until you want to take the gold over some of the margins and then use No. 60 gold. I prefer cutting out a piece, as though you were going to make a gold matrix, for a porcelain inlay. Cut out a piece of gold large enough to cover the entire margin of the cavity and force it into the cavity and fasten it to the gold you have already packed into the cavity. Now, if you want to use a matrix, your No. 60 gold has been carried over the gingival margin, and you may adjust your matrix and bring it up as tight as you like. You have got No. 60 gold covering the margin of the cavity everywhere. Thoroughly and carefully burnish, and there are no air spaces. It is the most perfect joint that can be possibly made by human skill, and then you begin to pack your gold into that, and then everywhere you have No. 60 gold for the finish lying over the edges of the cavity.—Dr. J. Leon Williams in *Brief*.

*Dental Caries.* From the standpoint of treatment, caries of dentine has no especial importance except the weakening of the tooth, exposure of the pulp, etc., caries of enamel is the initial lesion. Decay of dentine is secondary and will occur only after decay has penetrated the enamel. It is the study of caries of enamel, the local conditions of its beginning, the conditions giving opportunity for its spreading in certain directions, the limitations of spreading, etc., that are important to the practitioner.—G. V. Black in *Review.*

*Polishing the Proximal Surfaces of Fillings.* The problem of polishing the proximal surfaces of gold or other fillings is often difficult at the best. A plan that has afforded me a great deal of satisfaction where teeth were crowded, and when it was desirable to use the polishing strip, was to take the scissors and cut the end of a suitable strip to a point and then to make a hole about half an inch from the end for the insertion of dental floss. A convenient length of floss pulled through six or seven inches, the two ends laid together, the strip thus engaged can be readily drawn between the teeth by first slipping the floss between in the usual way from the occlusal surface and then pulling the attached strip either towards the buccal or lingual side. When the point is in sight it can often be pulled far enough with the cotton carriers to enable the operator to grasp it with the fingers. It is always advisable to coat the strip on both sides with a lubricant, as vaseline.—Dr. E. B. Lodge in *Dental Review.*

*The Period to Start Dental Operations.* I prefer to start with the eruption of the first teeth. My idea is that the child's teeth should be kept under constant surveillance by the dentist from that day to the age of maturity and as long thereafter as may be desirable. I would begin to care for the teeth and the child's mouth as soon as any interference was indicated. One of the newer facts made known to us is that the deciduous set of teeth are often in malocclusion and the jaws and facial bones are undeveloped or malformed, and that this occurs at the earliest stage of development. Here is a place of beginning that the large majority of dentists do not even know anything about and consequently give it no attention whatever. Volumes almost have been written as to advice to be given to pregnant women, but here is a much more important matter that is and has been almost wholly neglected. There is no question in my mind as to the practicability of carrying any healthy baby to full maturity without the loss of a single permanent tooth and with every tooth in full occlusion, with little or no destruction by caries, and the peridental tissue in perfect health.—N. S. Hoff in *Review*.

*High Pressure Anesthesia.* If you would succeed with the high-pressure syringe, carefully observe the following rules:

First: Never use it in a carious cavity, mainly for two reasons—because (*a*) the mouths of the dentinal tubuli are filled with debris, therefore you will not be likely to succeed, and (*b*) if you do succeed in desensitizing the pulp you will probably force ptomaines into it which will be almost sure to cause its death later. If it does not die, it will probably give the patient trouble for which the cocain will get the blame.

Second: Always enter the tooth from some surface point, for by so doing you will have a definite idea as to the relative distance of the pulp. This knowledge is imperatively requisite, for when you recall the case of my patient here you will remember that much more time is required to get the desired result than if you were entering a lower incisor at the gingival line.

Third: Age and temperament must be carefully considered, particularly as to the time required for desensitizing. We know that the dentinal tubuli and pulp in young patients are much larger than in aged ones, therefore much less time is required to obtund their teeth. This is particularly true of the nervous and bilious temperaments as compared with the sanguine and lymphatic. The danger of injuring the pulp by the use of cocain, as suggested above is so remote that the ultra-conservatism which prevents its use spells injustice to the patient.—Dr. W. T. Jackman in *Dental Cosmos*.

*Matrices.* Here are two samples of matrix material, steel and German silver, and a model with two matrices in place. The matrices are drawn very tight and the gingival margin and the silk with which they are threaded should be wrapped once, twice or thrice around the tooth as far down as seems desirable for the case at hand. The adjustment of these matrices is as follows: A piece of steel is cut suitable to the case and a hole punched in each end close to what will be the gingival border. A piece of floss about eighteen inches long is passed through the hole in one end, drawn across the matrix and through the other hole. Take a knife, or the end of an instrument, and test them; you will find them quite satisfactory. The matrix should then be passed between the teeth, passing both ends of the silk two or three times in opposite directions around the tooth to be filled, and tightly tied. On account of the tendency of the ligature to seek the smallest circumference when tightly tied, this procedure pulls the edge of the matrix below the gingival margin and makes it very tight at that point. The rest of the matrix may be wrapped with silk, little or much, according to the varying circumstances of the case. This method used with the exceedingly thin steel, makes a very good gingival margin, and allows a sufficient knuckling of the filling with practically no weighing. The distance of the holes from the gingival border of the matrix is important, and of course, should be according to the case in hand.

Punching the steel is somewhat difficult, but a good punch may be made by grinding the end of an old instrument square and hammering the steel with it on a copper cent on an anvil, and using the instrument with a hammer. The steel is very hard to cut smoothly with a rubber dam punch, which I use for the German silver. I have not been able to get steel as thin as this here, but have imported this through my clock repairer—Dr. K. C. Smith in *Journal of Allied Society*.

*Revocation of License to Practice.* In this issue appears a paper by O. J Cunningham, London, Ont., advocating the disciplining of dentists who do not observe asepsis in their practices, or who are alcoholic, drug habituates or insane. The arguments in support of action are irrefutable. The only question that might delay action is the power of the Board. But if the Board has the power to discipline members for unprofessional conduct and may hinder the incompetent from entering the profession, surely they have the power to say that a dentist who does not practice in an aseptic manner, or is habitually drunk or under the influence of drugs, or insane, is guilty of unprofessional conduct and is incompetent. and besides this is a menace to the public.

There is no reason why by-laws might not be passed to deal with these important questions if need be. The mere presence of such by-laws might have the effect of more care in practice, less drunkenness and fewer drug habituates. What better illustration could we have of our duty to the public than by-laws prohibiting the incompetent, degenerate or insane from continuing to be a menace to public health, even if they had obtained licenses. Because a young man has shown himself competent, wise and of a good moral character before an examining board, it does not follow that he will always be so. The Board which has the power to say that a man has the proper qualifications to begin the practice of dentistry should have the power to say that he has the qualifications to continue.

Lest the Board might think that this discussion has little to warrant it in practice, let any member visit any of a dozen offices that might be named at a moment's notice, and if he is not convinced that more care is needed in ordinary cleanliness, then this article is wide of the mark. Within a week examples of the dangers to the public from drunkenness, personal unfitness, drug habituates and the insane have come to the writer's attention. There is a drug habituate in this province with a license to practice dentistry who is a real danger to the public where he is located. Patients may come under his care when he might suddenly become overpowered by a dose of cocain, which he uses with a hypodermic, and not be able to give assistance that would save their lives. There is a semi-lunatic with a license to practice in Ontario. This man occasionally has visions and a command from the Lord. On one occasion he had a commond to extract all of a patient's teeth, but owing to the quick-wittedness of the patient an accident was averted. The provincial secretary's department says there is no means of dealing with such a case unless some one is ready to have him committed for trial on a charge of insanity. This man might be sane enough to do many kinds of work, but he should not have the privileges granted by license to practice dentistry. This case should be investigated. After an accident occurs it would be too late to avert it by taking away his license.—*Dominion Dental Journal.*

